# Detection of Somatic Mutation in Exon 12 of DNA Polymerase β in Ovarian Cancer Tissue Samples

**DOI:** 10.29252/ibj.22.5.355

**Published:** 2018-09

**Authors:** Kalyani Khanra, Indranil Choudhuri, Nandan Bhattacharyya

**Affiliations:** Department of Biotechnology, Panskura Banamali College, Panskura RS, PIN 721152, Purba Medinipur, West Bengal, India

**Keywords:** DNA polymerase β, Ovarian cancer, Single nucleotide polymorphism

## Abstract

**Background:**

DNA polymerase β (pol β) is a key enzyme of base excision repair pathway. It is a 1-kb gene consisting of 14 exons. Its catalytic part lies between exon 8 and exon 14. Exon 12 has a role in deoxyribonucleotide triphosphate selection for nucleotide transferase activity.

**Methods:**

Genomic DNA was isolated from ovarian carcinoma samples. Single strand conformation polymorphism method was used to detect mutation in genomic DNA.

**Results:**

Twenty-four patients of the 152 pair of tumor samples (15.8%) exhibited a point mutation (C→G) in position 725 in exon 12, which shifts proline to arginine (P242R). Statistical analysis showed a significant (*p* < 0.001) relationship between pol β mutation and the age of detection.

**Conclusion:**

This is a newly reported somatic mutation of pol β in ovarian carcinoma patients from India.

## INTRODUCTION

As estimated by American Cancer Society, around 22,400 women will be diagnosed with ovarian cancer in 2017, out of which 14,080 patients may be deceased[1]. Ovarian cancer ranks fifth in cancer deaths among women[1]. Mortality due to ovarian cancer may exceed 68% in women. In general, 80-90% of all ovarian tumors are sporadic, whereas the rest are hereditary. Also, DNA repair gene has a role in cancer formation[2].

DNA polymerase β (*pol β*) is a DNA repair gene that is the major player of base excision repair (BER) pathway[3]. It protects DNA from oxidative stress[4,5] and alkylating agent[6]. Human pol β is encoded by a 34-kb single-copy gene, located in the short arm of chromosome 8 (8p12-p11)[3]. The catalytic sub-domain coordinates two divalent metal cations that assist the nucleotidyl transferase reaction[7-9]. BER is divided into two sub-pathways, in which one single nucleotide is repaired by short patch pathway, whereas 1-6 nucleotide(s) is/are incorporated during the repair process of long-patch repair mechanism[10]. At least 30% of human tumors studied express pol β variant proteins that are not present in normal tissue[11]. Single amino acid substitutions are found in 48% of these tumors, 12% contain truncated variants,14% harbor multiple alterations, and 25% express a protein in which exon 11 is deleted through alternative splicing[12]. It has previously been reported that expression of cancer-associated pol β variants in mouse cells could lead to a series of cancer-associated phenotypes, including an increased mutation frequency and the induction of cellular transformation[13]. Pol β is a key enzyme in DNA repair, and any perturbations in its expression or function can result in increased mutation frequency and genomic instability[3]. Therefore, the present project was designed to screen the mutation of *pol β* gene in the catalytic region in the ovarian carcinoma samples.

## MATERIALS AND METHODS

### Sample collection

A total of 173 samples along with their normal counterparts were collected from hospital and nursing homes of Haldia, West Bengal, India, as per the ethical clearance by the Institutional Ethical Committee of Panskura Banamali College, Panskura, West Bengal, India. Of these samples, 152 were sporadic and the rest were hereditary. Samples were characterized on the basis of age of detection, stage, and type of cancer. Also, informed consents were collected from all the patients.

### Preparation of total genomic DNA

Tumor samples were frozen in liquid nitrogen and crashed to prepare powder. Genomic DNA was then isolated from these powdered samples[14,15]. In brief, samples were incubated at 56°C for 10-12 h in DNA lysis buffer containing 100 mM of NaCl, 100 mM of Tris-HCl (pH 8.0), 20 mM of EDTA (pH 8.0), 0.5% of SDS, and 0.1 of mg/ml proteinase K, followed by centrifugation at room temperature at 7500 ×g for 15 minutes to collect supernatant. The aqueous solutions were extracted with phenol/chloroform. DNA from the supernatant was precipitated by using 1/2 volume of 7.5 M ammonium acetate and 2 volumes of 100% alcohol. DNA concentration and its purity were measured by spectrophotometry, and DNAs were stored at -80 °C for future use.

### Polymerase chain reaction (PCR)

PCR was used to amplify the exon specific coding sequence of the *pol β* gene. PCR reactions were performed with the primer set FP (5’-TGGCCTTGTGTTTTACTTGATTAA-3’) and RP (5’-TTGGCAAGAACATATGGCTCTT-3’) in a total volume of 25 μl using an Eppendorf thermocycler. The reaction mixture contained 50 ng of template DNA, 0.25 mM of each primer, 2.5 µl of 10× PCR buffer (100 mM Tris-Cl, 100 mMKCl, (NH_4_)_2_SO_4_, 2.5 µl of 15 mM MgCl_2_, pH 8.0), 0.25 mM of dNTPs, and 1 unit of Pfu DNA polymerase[9]. The thermo-cycling profile was set at 94 °C for 3 min, followed by 30 cycles of a denaturing step at 94 °C for 30 seconds, an annealing temperature at 55 °C for 30 seconds, an extension step at 72 °C for 30 seconds, and a final extension at 72 °C for 5 min. The PCR products were electrophoresed on a 1.2% agarose gel to detect the desire product and were purified using GENECLEAN^®^ Kit (Qbiogene Inc., Carlsbad, California, USA) according to manufacturer’s recommendations. Purified PCR products were used for single strand conformation polymorphism (SSCP).

### Detection of polymorphism

SSCP were designed to evaluate polymorphisms at single loci. PCR products were heated at 95 °C for 5 minutes in the presence of 1 mM of NaOH solution to form single-stranded DNA[10,11] and rapidly cooled on ice. The mobilities of the single-stranded fragments were compared by electrophoresis on a polyacrylamide gel with wild-type pol β PCR products. Bands were detected using SYBR^®^ Gold (Invitrogen Life Science Technologies, USA). Any PCR product showing heterogeneity rather than normal bands were cut from the gel, purified and sequenced by commercial facility (Chromous Biotech Pvt. Ltd., Bengaluru, India).

### Statistical data analysis

All the statistical analyses were performed with JASP (version 0.8.3.1). Statistical analyses were conducted using the free software JASP using default priors. We reported Bayes factors expressing the probability of the data given H1 relative to H0 (i.e., values larger than 1 are in favor of H1)

## RESULTS

The histopathology results showed that 96 (63.2%) out of 152 samples were serous type, whereas 19 samples (12.5%) were either clear cell type or endometrioid type. In addition, 18 (11.8%) samples were mucinous. We also found that 15 (9.9%), 44 (28.9%), 40 (26.3%), and 53 (34.9%) samples belonged to the stages I, II, III, and IV, respectively. However, 17 (11.18%), 48 (31.57%), 48 (31.57%), 32 (21.05%), and 7 patients (4.6%) belonged to the age groups of 21-30, 31-40, 41-50, 51-60, and 61-70 years old, respectively. The average age of the patients was 42.95 years with minimum age of 24 years and the maximum age of 69 years with standard deviation of 10.07.

**Fig. 1:**
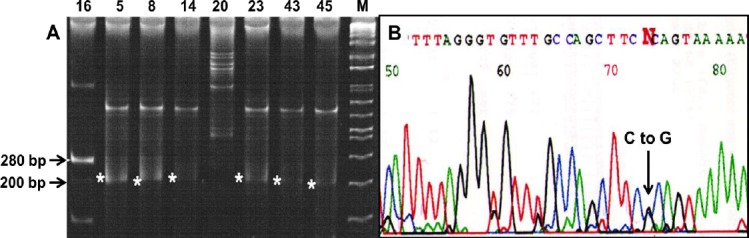
Detection of mutation. (A) SSCP analysis of exon 12. The expected size is 280 bp. The numbers above the lanes denote the sample number. M stands for DNA molecular marker (Fisher BioReagents™ exACTGene™ DNA Ladders). (B) Sequencing chromatogram. The chromatogram showed heterozygous mutation where one allele has been mutated from C to G.

The expected size of the PCR product was 280 bp (Fig. 1A). In this Figure, sample numbers 5, 8, 14, 23, 43, and 45 showed variation. The heterozygous mutation causing the change of C to G was identified at nucleotide position 725 of exon 12, obtained from tumor DNA of some patients. This is a missense mutation (C to G) as there would be a change in the amino acid (Fig. 1B). Of 152 samples, 24 showed synonymous mutation where change from amino acid proline to arginine (C to G) took place in exon 12. This is the newly report of somatic mutations of *pol β* gene in ovarian cancer.

Pearson correlation value (0.263) indicated a significant correlation (*p* < 0.001) between exon 12 mutation and the age at diagnosis of the patients where lower 95% credible interval (CI) and the upper 95% CI values are 0.107, and 0.402, respectively (Table 1). As indicated in this Table, a significant relationship was found between the stage of the tumor samples–type of the tumor samples and age at diagnosis of patients and stage of tumor samples.

**Table 1:** Pearson correlations

Bayesian pearson correlation	Exon	r	BF_10_	95% CI	

				Lower	Upper
Age at detection	12	0.263	40.564	0.107	0.402
Stage	12	-0.048	0.067	0.002	0.151
Type	12	0.030	0.14	0.003	0.201

For all tests, the alternative hypothesis specifies that the correlation is positive.

To determine any positive correlation between two variables and to determine whether the results were in favor of alternative hypothesis, Bayes Factor 10 (BF_10_) was tested. The observed correlation between the two variables (r)-age at diagnosis and the mutation of exon 12 was 0.263 and the associated base factor was 40.564 in favor of the alternative hypothesis. It indicates the alternative hypothesis predicted the data 40.564 times better than the null hypothesis, with a prior value of 1 (default) and the credible interval between 0.107 and 0.402.

**Fig. 2:**
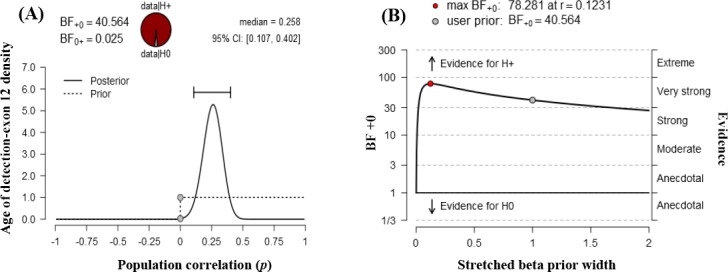
Statistical analyses. (A) Prior and posterior distribution analysis. (B) Bayes factor robustness analysis.

In Figure 2A, prior and posterior distribution are shown in dashed and solid line, respectively. Most of the posterior distribution values falls between 0.107 and 0.402. The dots in the plot represent the height of the curves of the null hypothesis of no correlation. The first dot on the prior distribution is higher than the dot of the posterior distribution, which means the BF supports the alternative hypothesis.

The robustness of the result was checked with a range of priors. Figure 2B is the graphical representation of BFas well as 95% CI for the correlation. In this graph, x axis represents the prior widths and y axis represents the value of the BF. BF above 1 indicates the evidence in favor of the alternative hypothesis, and the BF below 1 shows the evidence in favor of the null hypothesis. Here, the BF did not change too much unless the prior width was very small. The most value of the prior width of BF was above 10, which depending on the context can usually be considered as fairly strong evidence. The qualitative conclusion does not change with reasonable variations to the prior width; hence, the result is fairly robust.

The correlation coefficient (r) between stage and the mutation of exon 12 was -0.048 and the associated base factor was 0.067 in favor of the null hypothesis. The correlation coefficient (r) between type and the mutation of exon 12 was 0.03 and the associated base factor is 0.14 in favor of the null hypothesis. Both were not significant.

## DISCUSSION

Pol β is a 39-kDa protein of two subunits, one small subunit of single-stranded DNA-binding activity and dRPase activity and one large subunit of 31-kDa protein of catalytic activity[16]. The 1-kb *pol β* gene contains 14 exons[17]. Large subunit lies in exon 8 to exon 14. Exon 12 is located in the thumb region of catalytic part and has a role in dNTPs selection with nucleotide transferase activity[7,8]. Hence, mutation detected in exon 12 may influence catalytic activity if it expressed in mRNA. It has already been detected that DNA pol β has mutation, splice variant in tumor tissue sample. In 30% cases of cancer pol β mutation was found[11]. Various point mutations in cDNA were also detected, which influence the catalytic activity of DNA pol β[18-20].

In the present study, we first report the expression of altered product in genomic DNA of pol β in ovarian carcinoma tissue using SSCP. Our results demonstrated an association between pol β mutation and cancer cell, though there was a tendency of occurring mutation in serous sample and at age group of 51-60. As the first study to analyze the association between pol β alterations and individual susceptibility for development of ovarian cancer in a small Indian population, our results suggest that further verification with a larger population may be necessary to determine mutation probability. In addition, as proline residue causes a turn in the mender region, the change of amino acid from proline to arginine may alter the conformation of the pol #x03B2; DNA. The proline side chain is very non-reactive, whereas the aspartic side chain is very active. Therefore, it is important to study the role of this mutation in the future.
